# When two bleeds collide: Pituitary apoplexy masking a ruptured intracranial aneurysm

**DOI:** 10.4102/sajr.v30i1.3479

**Published:** 2026-07-14

**Authors:** Tavonga P.C.S. Bayela, Suzanne O’Hagan, Christelle Ackermann

**Affiliations:** 1 Division of Radiodiagnosis, Faculty of Medicine and Health Sciences, Stellenbosch University, Cape Town, South Africa

**Keywords:** pituitary apoplexy, intracranial aneurysm, subarachnoid haemorrhage, CT angiography, MRI, digital subtraction angiography

## Abstract

Concurrent pituitary apoplexy and ruptured intracranial aneurysm are rare, but potentially fatal, if unrecognised. A 53-year-old hypertensive woman presented with acute confusion and transient hemiparesis. Brain CT demonstrated a haemorrhagic sellar–suprasellar mass with atypical subarachnoid and intraventricular haemorrhage. CT angiography (CTA) confirmed a ruptured right middle cerebral artery (MCA) aneurysm and two additional unruptured aneurysms. Subsequent endovascular coil embolisation was successful. The pituitary macroadenoma was managed conservatively.

**Contribution:**

Early recognition using haemorrhage pattern analysis and CTA is critical to avoid missed dual pathology in pituitary apoplexy and to guide timely management.

## Introduction

Pituitary adenoma (PA) apoplexy and ruptured intracranial aneurysm (IA) are individually common, potentially life-threatening neurosurgical emergencies. Their simultaneous occurrence is rare. Both conditions may present with overlapping clinical features, and a coexisting ruptured IA may be overlooked in patients with radiological evidence of pituitary apoplexy. Previous reports have emphasised this diagnostic pitfall. This case highlights the importance of careful haemorrhage pattern analysis on CT and early CT angiography (CTA) to detect concurrent aneurysmal rupture.

## Ethical considerations

Ethical clearance to conduct this study was obtained from the Health Research Ethics Committee (HREC) of Stellenbosch University (No. C26/02/005).

## Patient presentation

A 53-year-old female, known with chronic hypertension (defaulted on treatment), presented with a sudden onset of confusion and transient right-sided weakness that spontaneously resolved. On examination, the patient had an elevated blood pressure of 240/132 mmHg and was confused.

**FIGURE 1:**
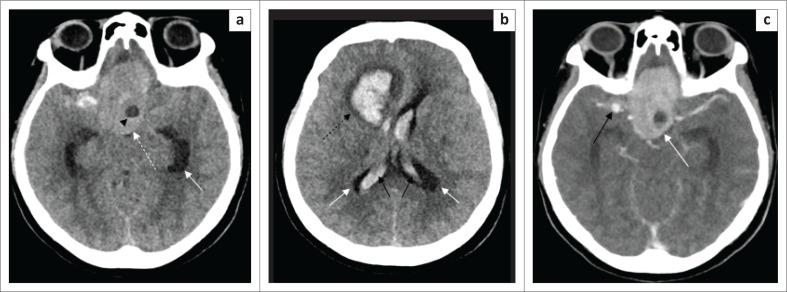
(a, b) Non-contrast CT axial images at the suprasellar cistern (a) and lateral ventricles (b) demonstrating a heterogeneous sellar-suprasellar mass (white dashed arrow) with cystic components and acute intralesional haemorrhage (arrowhead). Note the right frontal parenchymal haemorrhage (dashed black arrow) adjacent to the mass, intraventricular haemorrhage (black arrows) and obstructive hydrocephalus (white arrows). (c) Contrast-enhanced axial CT image demonstrating the enhancing sellar-suprasellar mass (white arrow) and a focal bright contrast collection in the right MCA cistern (black arrow), suspicious for a saccular aneurysm.

Initial pre- and post-contrast CT brain revealed an avidly enhancing intrasellar mass with cystic areas and acute intralesional haemorrhage (Figure 1a). The mass measured 50 mm × 32 mm × 24 mm (anteroposterior × transverse × craniocaudal) and extended through the diaphragma sellae into the suprasellar region. Additional findings included a right frontal perilesional haematoma, subarachnoid haemorrhage (SAH) in the right middle cerebral artery (MCA) cistern with subfalcine herniation and widespread intraventricular haemorrhage with obstructive hydrocephalus (Figure 1b). These were initially interpreted as extensions of tumour-related bleeding. However, on review of the images, the extent and distribution of the extra-tumoural haemorrhage raised suspicion for a co-existing ruptured aneurysm. On further scrutiny, an aneurysmal dilatation of the right MCA was discovered on the same images (Figure 1c).

**FIGURE 2:**
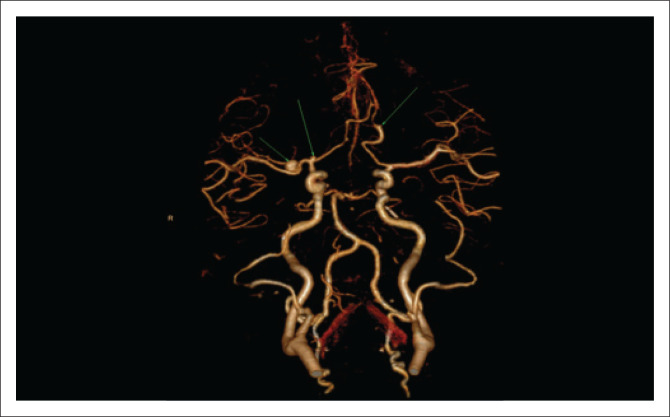
3D reconstructed maximum intensity projection (MIP) of CTA showing multiple saccular aneurysms, with the largest at the right MCA bifurcation and two smaller aneurysms at the right terminal ICA and A2 segment of the left ACA (green arrows).

CTA confirmed a ruptured aneurysm at the right MCA bifurcation and identified two additional aneurysms at the right terminal internal carotid artery (ICA) and A2 segment of the left anterior cerebral artery (ACA) (Figure 2).

**FIGURE 3:**
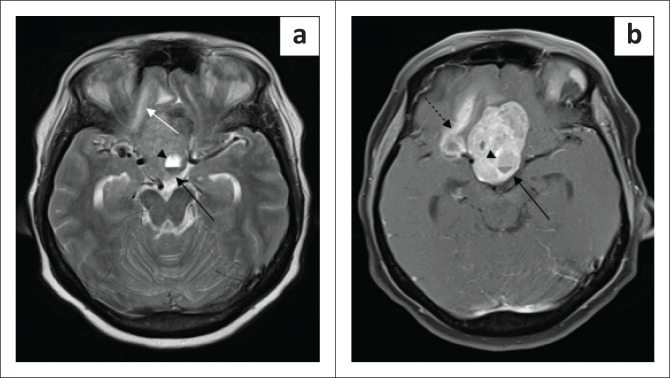
(a, b) Axial T2-weighted and post-contrast T1-weighted MR images showing the heterogeneous sellar-suprasellar mass (black arrows) with T2W hyperintense acute intralesional haemorrhage and fluid levels (arrowheads), and enhancing solid components. Surrounding T2 hyperintensity around the right frontal haematoma (white arrow) is consistent with vasogenic oedema. Peripheral enhancement of the haematoma on post-contrast T1-weighted images (dashed arrow) reflects blood-brain barrier disruption secondary to acute haemorrhage.

Subsequent MRI better characterised the lesion as a heterogeneous mass with cystic areas containing fluid levels and acute intralesional haemorrhage - hyperintense on T2-weighted images (Figure 3a). The solid component demonstrated contrast enhancement, with oedema around the right frontal haematoma (Figure 3b). Laboratory investigations confirmed panhypopituitarism.

The final diagnosis was PA apoplexy with concurrent ruptured right MCA aneurysm – the presumed source of SAH based on the haemorrhage distribution in the MCA cistern. The largest aneurysm was successfully treated with emergent digital subtraction angiography (DSA) and endovascular coil embolisation. The pituitary apoplexy was managed conservatively. The patient improved and was discharged for outpatient endocrine and neurosurgical follow-up with interval MRI.

## Discussion

Coexisting IA and primary brain tumours are not uncommon, and PA is one of the tumours most highly associated with IA, with an incidence of coexisting IA ranging from 3.5% to 8.3% – significantly higher than the 2% – 4% incidence of IA in the general population.^1,2^

The underlying pathophysiology of IA in a patient with PA is multifactorial and involves both direct and indirect effects of the PA on the vasculature.^1,3,4^ Mechanical factors include tumour encasement of vessels in the circle of Willis (polygon of Willis), cavernous sinus invasion by the tumour and mass effect causing traction or compression of adjacent arteries.^1,2,3,5,6^ Cavernous invasion by itself is a significant risk factor for aneurysm formation and largely explains why the ICA is the commonest aneurysm location in these patients.^1,2,5^ Aside from directly weakening the vessel wall, cavernous invasion may also induce circulatory imbalance, which further compromises vascular wall integrity.^1,3^ Additionally, the compression or traction of vessels by the expanding tumour causes microanatomical alterations, which in turn modify local haemodynamics, thereby inducing aneurysms, not only in the encased vessels, but also in other vessels in the vicinity of the PA.^1,3,7,8^ Cavernous sinus invasion is particularly significant as it allows direct tumour contact with the ICA wall. This can result in local vessel wall weakening through tumoural invasion or chronic mechanical stress, while simultaneously inducing circulatory imbalance with turbulent flow and altered shear stress on the arterial wall. These haemodynamic changes promote endothelial injury and focal outpouchings that evolve into aneurysms.^1,3,5,6^

Hormonal factors also contribute. The majority of PAs are non-functioning (NFPAs); however, of the secretory PAs, growth hormone-secreting (somatotroph) adenomas show a particularly strong association with IA.^1^ GH and IGF-1 are known to trigger neovascularisation and induce degenerative changes by decreasing type III collagen within vessel walls.^1,5,6,9,10^ These changes not only increase susceptibility to aneurysms, but also contribute to increased local circulation, further altering the haemodynamics.^1,11^

These combined effects explain why associated IAs are typically located in the anterior circulation and are either encased by tumour, in contact with it, or in close proximity.^1,6^ In most cases, the aneurysms are solitary, and there is an inverse relationship between the number of aneurysms and their incidence of occurrence.^1^ Interestingly, the presented patient had three aneurysms, all located in the anterior circulation, as expected.

While it is established that patients with PA are prone to IA, these aneurysms are commonly discovered incidentally during imaging of the PA, usually unruptured at the time.^1,2,5^ Acutely ruptured IA presenting concurrently with pituitary apoplexy in the same patient is exceedingly rare, and to the authors’ knowledge, only seven cases, including the present report, are documented in the literature.^12,13,14,15,16,17^

The simplest explanation for this rare occurrence is that an aneurysm encased by or in close proximity to the PA may rupture directly into the tumour, resulting in intratumoural haemorrhage. In a case reported by Yoshida et al., active contrast extravasation from a ruptured aneurysm encased within the PA was demonstrated on dynamic post-contrast MR imaging. The authors proposed that the IA rupture increased intratumoural pressure, which in turn compressed pituitary vessels and resulted in secondary apoplexy.^16^ In several of these cases, aneurysm rupture either triggered secondary apoplexy or occurred concurrently, with haemorrhage extending into the tumour, suprasellar cistern or ventricles.^6,12,15,16^

A common thread amongst most of these cases, as in the presented case, is that the dual pathology was missed on initial imaging and only made upon review of initial imaging or subsequent imaging.^12,13,14,16,17^ In these cases, the aneurysm was usually the overlooked component.^12,13,14,16,17^ Several factors contribute to this diagnostic pitfall. Firstly, the two have overlapping clinical symptoms. Secondly, haemorrhage from the pituitary apoplexy may extend into the suprasellar cistern, ventricles and/or frontal lobe, leading to the natural presumption that all bleeding originates from the tumour. Thirdly, catheter angiography – the gold standard for diagnosing IA – is not routinely performed in the workup of pituitary apoplexy.

In the present case, the distribution of haemorrhage, particularly the focal collection in the right MCA cistern, was atypical for isolated pituitary apoplexy and provided the key clue to the dual pathology. This highlights the common diagnostic pitfall of premature attribution of all haemorrhage to the pituitary lesion because of overlapping clinical and imaging features.^12,13,14,16,17^

From a management perspective, securing the aneurysm first (whether ruptured or unruptured) is recommended. The pituitary mass may provide structural support to an adjacent or encased aneurysm; therefore, tumour debulking or removal, risks catastrophic haemorrhage from the IA.^4,9,12^ It is therefore crucial to diagnose concurrent IA prior to any pituitary surgery. In this case, successful endovascular coil embolisation of the ruptured right MCA aneurysm allowed conservative management of the pituitary apoplexy with a favourable outcome.

This rare dual pathology underscores the need for a high index of suspicion and multidisciplinary collaboration between radiologists, neurosurgeons and endocrinologists when managing complex sellar lesions with atypical haemorrhage patterns.

### Key radiological learning points

- Extra-tumoural haemorrhage, especially focal SAH remote from the sellar or disproportionate intraventricular/frontal haematomas, should prompt urgent vascular imaging.^12,14,17^
- Contrast-enhanced CT may reveal subtle aneurysmal contrast pooling; careful scrutiny of the circle of Willis and perisellar vessels is essential.
- CTA with 3D reconstructions is highly valuable for confirming multiple aneurysms and guiding endovascular planning.
- MRI excels at characterising the pituitary lesion (including assessment of cavernous sinus invasion) but cannot reliably exclude vascular pathology.

## Conclusion

Extensive extra-tumoural haemorrhage in pituitary apoplexy should raise suspicion for a coexisting ruptured IA. This case highlights the critical importance of meticulous review of initial CT images and the liberal use of CTA when haemorrhage distribution is atypical for isolated apoplexy. Early recognition of this rare dual pathology is essential to prevent diagnostic delay, avoid potentially catastrophic haemorrhage during pituitary surgery, and enable timely endovascular intervention. Radiologists play a pivotal role in identifying this life-threatening combination through vigilant image interpretation and a high index of suspicion.
